# The First Case of Cribriform-Morular Thyroid Carcinoma and FAP with APC Gene Mutation in China: A Case Report and Brief Review

**DOI:** 10.1155/2023/6222432

**Published:** 2023-05-18

**Authors:** Sucong Lyu, Guoqiang Zhong, Hui Chen, Jin Li, Mingsong Li

**Affiliations:** ^1^Department of Gastroenterology, The Third Affiliated Hospital of Guangzhou Medical University, Guangzhou, China; ^2^Department of Pathology, The Third Affiliated Hospital of Guangzhou Medical University, Guangzhou, China

## Abstract

The cribriform-morular variant of papillary thyroid carcinoma (CMV-PTC) is now designated as morular cribriform thyroid carcinoma (CMTC) according to the 5^th^ edition of the World Health Organization (WHO) Classification of Thyroid Tumors. CMTC can appear within a familial adenomatous polyposis (FAP) or be sporadic. We report the first case of a young female patient in China who was diagnosed with FAP and CMTC with a mutation in exon 16 of the APC gene underlying the disease. The main purpose of this case report is to provide a special pathological type of thyroid tumors, which is expected to be helpful for clinical work in the future.

## 1. Introduction

CMTC was first described as a distinctive variant of papillary carcinoma representing the sporadic counterpart of familial adenomatous polyposis-associated thyroid carcinoma by Cameselle-Teijeiro and Chan in 1999 [1]. Subsequently, in the 4^th^ edition of the WHO Classification of Tumors, both the sporadic and familial forms (associated with FAP) of this tumor were considered as the cribriform-morular variant of PTC [2]. CMTC is an extremely rare histological type, representing approximately 0.16% of all thyroid malignancies [3], and it is considered a distinctive entity associated with permanent activation of the Wnt/beta-catenin pathway [4]. Immunohistochemical staining showed diffuse expression of cytoplasmic and nuclear *β*-catenin while the thyroid transcription factor-1 (TTF-1) protein was only in the cribriform areas [5]. Due to the lack of markers of follicular differentiation, such as the lack of positivity for thyroglobulin (TG) and negativity or weak staining for PAX8, CMTC was reclassified as tumor of uncertain histogenesis according to the new WHO classification [5]. FAP is an autosomal dominant polyposis syndrome, characterized by hundreds of colorectal adenomatous polyps. To our knowledge, there are few reports about CMTC and FAP with APC gene mutations. The literature shows that [6] CMTC accounts for approximately 0.2% of all papillary thyroid carcinoma, and approximately 63% of the cases were reported in Asia, suggesting that this tumor was more common in Asian populations. However, information about APC mutations associated with CMTC in reported cases in Asia remains scarce.

Here, we present the first case of CMTC and FAP with a gene mutation in exon 16 of the APC gene in China. She was doing well after the treatment and was followed up for 6 years.

## 2. Case Report

A 27-year-old Asian female presented to our surgical department with a thyroid goiter of 12-month duration and recent onset of esophageal foreign body sensation. She had a personal history of FAP and a previous laparoscopic right hemicolectomy 5 years ago. There was no significant medical or other surgical history of note and no prior history of head and neck irradiation. This patient also has a strong family history of FAP: her grandfather, uncle, and younger brother had a history of intestinal polyps, and her mother and aunt died of colon cancer. Although there was a family history of colon cancer, there was no family history of thyroid cancer. Physical examination revealed thyroid follicular nodular disease.

**Figure 1:**
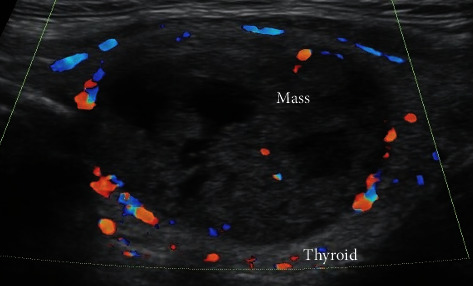
Ultrasonographic finding of the left thyroid nodule (28 mm × 19 mm in size).

Thyroid function tests and calcium levels were within normal limits. An ultrasound examination of the thyroid demonstrated a 4 mm × 3 mm nodule in the right thyroid lobe, two nodules in the left thyroid lobe, the smaller one measuring 4 mm × 4 mm in size and the larger one measuring 28 mm × 19 mm in size, which was classified as an ACR TI-RADS 5 nodule (Figure 1).

Gastroscopy showed multiple polyps in her stomach, and histological examination confirmed that they were fundic gland polyps. Colonoscopy revealed multiple polyps throughout the colon that were proven to be villous tubular adenoma with high-grade intraepithelial neoplasia.

She underwent a subtotal thyroidectomy in the right thyroid lobe and a total thyroidectomy in the left thyroid lobe with lymph node dissection. The postoperative period was uneventful, and she was discharged 7 days after surgery.

**Figure 2:**
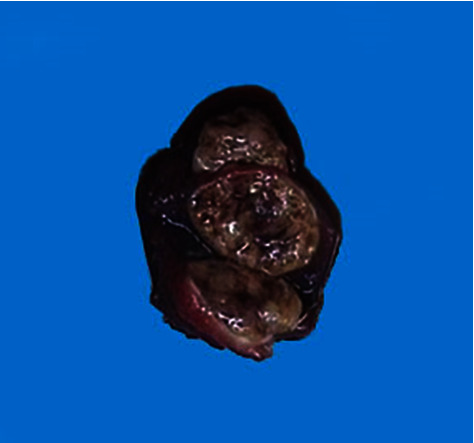
Macroscopic appearance of the thyroid lobe showing the cut surface of cribriform-morular thyroid carcinoma.

**Figure 3:**
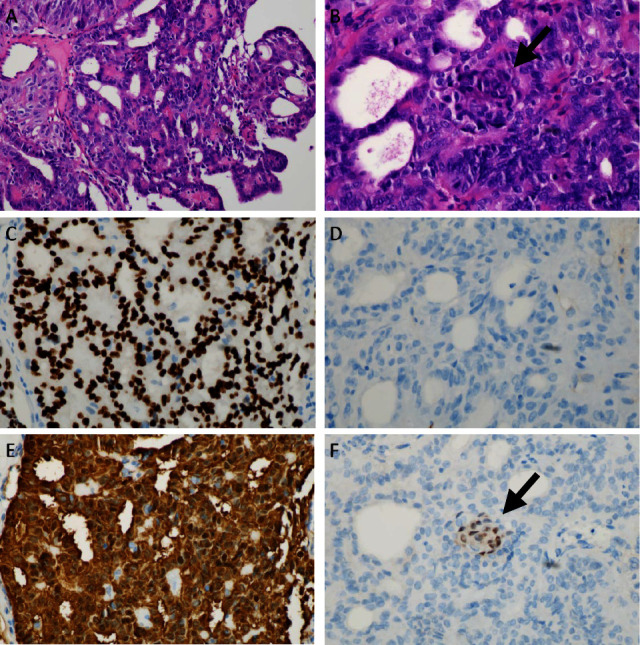
Histopathological evaluation of the thyroid carcinoma. (a) Cribriform and papillary growth pattern of tumor cells, low power. (b) Tumor area showing a cribriform growth pattern along with a morular structure (black arrow), high power. (c) TTF-1 was positive in nuclear of tumor cells, high power. (d) TG was negative in tumor cells, high power. (e) *β*-Catenin was positive in the cytoplasm and nucleus of tumor cells, high power. (f) CDX2 was positive in the nuclei of the morulae, high power.

Macroscopic examination of the left thyroid lobe showed one clean-edged nodule measuring 30 mm × 20 mm in size (Figure 2), while the right thyroid lobe showed no nodule. Histopathological evaluation showed the tumor cells arranged in a cribriform pattern in which the nuclei were enlarged and crowded, with nuclei grooves and abundant cytoplasm. Immunohistopathological staining for *β*-catenin showed diffuse nuclear and cytoplasmic staining; TTF-1 was positive in the nuclear of the tumor cells. CDX2 was positive in the nuclei of the morulae, while TG was negative in the nucleus and cytoplasm of the tumor cells (Figure 3).

Colonoscopy screening of her younger brother and two older brothers showed multiple polyps throughout the intestinal tract, and histopathological examination confirmed that were tubular adenoma while ultrasound of the thyroid demonstrated no nodule or thyroid carcinoma in their thyroid lobes.

The APC gene was sequenced for the female patient and her younger brother, and then we identified the same mutation (NM_000038.6): c.3183_3187del (p.Q1062^*∗*^) in exon 16 which confirmed the diagnosis of FAP. Unfortunately, her two elder brothers and other relatives refused to have the APC gene sequencing for financial reasons.

She was followed up for 6 years after thyroidectomy. No recurrence of CMTC was found, and the thyroid function tests were within normal limits. During the follow-up period, she underwent several times of colonoscopies and the histopathological examinations confirmed that multiple polyps were tubular adenoma.

## 3. Discussion and Conclusion

The APC gene is a tumor suppressor gene located on chromosome 5q22.2. The majority of APC mutations can lead to a truncated protein lacking *β*-catenin binding site, consequently, resulting in the storage of *β*-catenin [7]. CMTC is a rare morphologic entity often associated with FAP. Extensive research observed that around 39% of CMTC is associated with FAP [8], while another review shows that around 53% of patients with CMTC had FAP [6].

In the present study, we report the identification of the germline mutation of the APC gene, NM_000038.6: c.3183_3187del (p.Q1062^*∗*^), localized in exon 16, which was the first case reported in a young Chinese woman who was diagnosed with FAP and CMTC, while her younger brother was also diagnosed with FAP without thyroid carcinoma. This class 5 APC variant has previously been described as a germline variant in a FAP patient with PTC [9]. Codons 1020–1169 are reported to be the *β*-catenin binding site, which eventually leads to the development of CMTC [10], indicating the value of genotype-phenotype correlations in the prediction of this tumor. The genotype-phenotype correlation in Western FAP patients has been quite clear; however, further studies still need to be conducted in Chinese FAP patients [11].

Although previous studies found that the specific APC mutation site is likely to be associated with clinical manifestations [12], researchers [13] found that the genotype-phenotype correlation was not completely predictable, which indicated that the surveillance and management of FAP patients should be based on genotype, colonic phenotype, and family clinical history. We speculate that codon 1062 is responsible for the development of CMTC in this case, as it is one of the *β*-catenin binding sites. Since the female patient and her younger brother share the same gene mutation, further clinical follow-up is required.

CMTC is commonly seen in young females, and the female-to-male ratio is 31 : 1 [6]. The average age and median age of presentation for this tumor were 28 and 24 years, respectively. However, the mean age in male patients with CMTC was 43 years, and the difference in age of onset between males and females was significant [6]. The immunoreactive of ER*α* in the nucleus of this tumor is probably the key to the female preponderance of this disease [10]. Considering the gender and age of this patient's younger brother (18 years old), these may explain why the female patient had CMTC while her younger brother was negative for thyroid carcinoma.

Because of the presence of papillae and diagnostic nuclear features, this tumor was first classified as a subtype of papillary thyroid carcinoma. The tumor cells are cuboidal or columnar and sometimes spindle-shaped; tall cells and columnar cells may also be identified sometimes; thus, it is necessary to differentially diagnose tall cell variance and columnar cell variance of PTCs [8].

Recent studies found that CMTC did not display common biomarkers of papillary thyroid carcinoma, such as BRAF VE1 and HBME1, and rarely have RAS or PIK3CA mutations [5, 14]. However, researcher found that some patients had somatic “second-hit” mutations in APC, or somatic variants in other genes include KTM2D, KMT2C, CTNNB1, AXIN1, and RET/PTC rearrangements, which may cooperate with the APC germline mutation in FAP patients and act as a driver in thyroid cancer [14–16]. The correlation between the location of the germline mutation in APC and the somatic “second-hit” still needs further research. It is known that there are no other relatives with thyroid cancer in this family, and unfortunately, genome sequencing was not performed on this tumor.

In addition, these tumors show diffuse cytoplasmic and nuclear *β*-catenin expression due to the mutation of APC. In the nonmorular component, the tumor cells are always positive for TTF1 and are sometimes positive for PAX8 but always negative for TG [5]. While the morulae areas are positive for CD5, CDX2, and CK5, they lack TTF1 expression [5]. The cribriform areas also express estrogen and progesterone receptors. The above reasons lead to the reclassification of the tumor. The APC mutation and the immunohistochemical hallmark features of these tumors are of great value in diagnosis and differential diagnosis of CMTC and other PTCs.

The prognosis of the tumor is usually excellent if patients undergo endoscopy check and therapy for FAP as needed because CMTC is generally considered as an indolent disease. Total thyroidectomy is sufficient for the treatment of most tumors, and extensive lymph node dissection is generally unnecessary [6]. In this case, the female patient has been followed up for 6 years and showed no recurrence of CMTC, consistent with the good overall prognosis of most CMTC cases [4, 5].

In conclusion, CMTC is a rare type of thyroid tumor often associated with FAP. The APC mutation and immunohistochemical hallmark features of these tumors could differentiate them from other thyroid carcinomas. The genotype-phenotype correlation of Chinese FAP patients is not clear yet. Since the prognosis of CMTC is usually excellent, this rare but important thyroid carcinoma should be familiarised with, and thyroid examinations should be performed routinely in FAP patients.

## Data Availability

The data used to support this study are included within the article. Further inquiries can be directed to the corresponding authors.
